# Editorial: Dysmetabolism, Obesity, and Inflammation: Three Prominent Actors in the Drama of Major Neuropsychiatric Disorders

**DOI:** 10.3389/fnins.2016.00368

**Published:** 2016-08-19

**Authors:** Tommaso Cassano, Luca Steardo

**Affiliations:** ^1^Department of Clinical and Experimental Medicine, University of FoggiaFoggia, Italy; ^2^Department of Physiology and Pharmacology “V. Erspamer,” Sapienza University of RomeRome, Italy

**Keywords:** dysmetabolism, obesity, inflammation, neuropsychiatric disorders, Alzheimer's disease

A large and convincing body of evidence demonstrates that medical conditions associated with chronic inflammatory and immunological abnormalities may represent risk factors for development of neuropsychiatric disorders. Indeed obesity, dysmetabolic conditions, including diabetes, metabolic syndrome, and autoimmune diseases are frequently associated to the development of neurodegenerative and psychiatric illnesses. Recent data have demonstrated that inflammatory cytokines and adipokines can interact with multiple transmitter pathways, neuroendocrine function, synaptic plasticity, and neurocircuits relevant to behavior regulation. Therefore, further understanding of mechanisms linking obesity, dysmetabolism, and (neuro)inflammation to neurological and mental alterations may be especially relevant to the treatment and prevention for such debilitating diseases that represent a major public health concern.

Along this line, the current Research Topic provides appreciable evidence showing that activated inflammatory responses can undermine disease-relevant affective, emotional, social, and cognitive functions, so that inflammatory processes may be particularly relevant for the onset and progression of neuropsychiatric disorders.

The present volume, at which contributed eminent scholars includes 11 review articles, 1 original research article, and 1 commentary and was organized in the attempt to cover the whole field of the interrelationship between inflammation, dysmetabolism, and neuropsychiatric disorders.

To this regard, a very updated and comprehensive review article by Castanon et al. provided converging evidence showing that obesity might be associated with exacerbated neuroinflammation leading to dysfunction in vulnerable brain regions associated with mood regulation, learning, and memory (Castanon et al.). Their findings shed new light on the pathophysiological mechanisms responsible for brain disorders associated to obesity and suggest innovative strategies for the treatment of neuropsychiatric complications occurring in obese patients.

Recent data, reviewed by Theoharides et al. demonstrated that pro-inflammatory molecules and related signaling pathways impact on multiple neurotransmitter circuits leading to synaptic damage, neuronal loss, and the activation of other inflammatory participants (Theoharides et al.). In particular, the authors emphasized the role of inflammation in the onset of brain “fog,” which represents a constellation of symptoms that include reduced mental acuity and cognition, inability to concentrate, as well as loss of short- and long-term memory. Brain “fog” may occur in patients suffering from many neuroimmune diseases, systemic mastocytosis, or disorders of mast cells activation, with increase in interleukin-6 (IL-6), tumor necrosis factor (TNF) levels. Moreover, the authors reported that the flavone luteolin might improve attention in children with autism and in mastocytosis patients with brain “fog” by reducing serum levels of TNF and IL-6. Similarly, Muller et al. provided an update about the role of inflammation in the pathogenesis of schizophrenia and the influence of immune activation on brain neurotransmissions. The authors also suggested an immune-based therapeutic approach in a subgroup of schizophrenics in whom an immune dysbalance is associated with chronic inflammation (Muller et al.). To this regard, Bonnot et al. emphasized the association between hidden neurometabolic diseases and psychiatric disorders. According to the authors, schizophrenia-like symptoms are by far the most frequent psychiatric warning signs of neurometabolic disorders. These morbidities have a significant impact on the life expectancy of patients, since subjects with chronic psychoses show a 2–3-fold increased risk of death, mostly from cardiovascular and metabolic diseases (Bonnot et al.). Therefore, Ventriglio et al. suggested that clinicians should screen and monitor these comorbidities to reduce mortality rates among patients with psychosis (Ventriglio et al.).

Aging predisposes to diseases via several mechanisms that in part converge on inflammatory pathways. As age advances, the immune system undergoes a process of senescence accompanied by the increased production of inflammatory cytokines able to affect behavioral and cognitive performances. Deleidi et al. discussed the link between immune aging and neurological disorders describing how metabolic changes linked to the aging, immunosenescence, and inflammation can enhance the development of neurodegenerative and neuropsychiatric diseases. The authors argued that targeting age-associated (neuro)inflammation could delay the onset or slow disease progression (Deleidi et al.).

In this context, glial cells have been proved to exert a crucial role. In fact, a growing body of evidence shows the remarkable complexity of inflammatory mechanisms in Alzheimer's disease (AD) and how they are often driven by alterations of glial cells functioning. In particular, the review paper by Steardo et al. explored the dual role of astrocytes in the onset and progression of AD. The authors questioning the validity of the prevalent neuron-centric vision of neuropsychiatric diseases highlighted the role of non-neuronal cells in the pathogenesis and progression of many diseases, also suggesting that astrocytes could be considered as promising target for future AD therapies (Steardo et al.).

Recent human and preclinical studies provided convincing proof that AD is a degenerative disease associated with dysmetabolic aspects, including impairments in brain insulin responsiveness, glucose utilization, and energy metabolism (Barone et al., 2016). Given this background, the review paper by Bedse et al. focused on the role of insulin signaling in the deposition of neuritic plaques and intracellular neurofibrillary tangles (Bedse et al.). Moreover, the authors proposed intranasal insulin administration as potential therapeutics, since preliminary clinical trials have shown cognition improvement without significant side effects (Bedse et al.). Similarly, Lutz and Meyer discussed the potential role of the pancreatic peptide amylin as a link between metabolic and neurodegenerative disorders in general, and type 2 diabetes mellitus and AD in particular, emphasizing the possible influence of amylin in the amyloidogenetic process, and providing the rationale for an amylin-based strategy in the treatment of AD (Lutz and Meyer).

However, although the link between dysmetabolism, obesity, inflammation, and neuropsychiatric/neurodegenerative disorders was extensively investigated in adulthood, there was no convincing literature demonstrating a positive correlation between maternal dysmetabolism and child's mental health. In this perspective, Rivera et al. summarized studies highlighting maternal obesity as one of the major risk factors for the development of mental health disorders in the offspring. Moving also from investigations carried out with animal models of maternal obesity, they further discussed the potential mechanisms by which such a condition impairs the development of neuronal circuitry, even suggesting possible strategies for prevention and therapeutic intervention (Rivera et al.).

Besides environmental factors (e.g., diet), the epigenetic regulation of gene expression has emerged as a potential contributor to the susceptibility and development of obesity. To investigate the individual sensitivity to weight gain/resistance, the research paper by Cifani et al. studied the regulation of gene transcription of several hypothalamic neuropeptides involved in the control of energy balance in rats developing obesity or not, when fed with a high fat diet (Cifani et al.). The authors provided evidence of selective and time-dependent transcriptional regulation of target genes in obese rats compared to non-obese counterparts (Cifani et al.).

Among the hypothalamic neuropeptides, oxytocin seems to play a crucial role in the regulation of feeding, metabolism, and in several behaviors associated with neuropsychiatric disorders (Romano et al., 2013a,b). To this regard, a very comprehensive review article by Romano et al. examined the role of oxytocin in a variety of physiological and behavioral processes suggesting that future research will clarify whether oxytocin may represent a promising target for innovative neuropsychiatric treatments (Romano et al.).

Taken together, this special issue might provide scientists and clinicians with new insights into this emerging area of research and it might offer more cues for the development of promising therapeutic strategies against such devastating illnesses.

## Author contributions

All authors listed, have made substantial, direct and intellectual contribution to the work, and approved it for publication.

### Conflict of interest statement

The authors declare that the research was conducted in the absence of any commercial or financial relationships that could be construed as a potential conflict of interest.
